# Metagenomic Analysis of the Respiratory Microbiome of a Broiler Flock from Hatching to Processing

**DOI:** 10.3390/microorganisms9040721

**Published:** 2021-03-31

**Authors:** Kelly A. Mulholland, Monique G. Robinson, Sharon J. Keeler, Timothy J. Johnson, Bonnie W. Weber, Calvin L. Keeler

**Affiliations:** 1 Department of Animal and Food Sciences, University of Delaware, Newark, DE 19716, USA; kamulhol@udel.edu (K.A.M.); mgiselle@udel.edu (M.G.R.); keelersj@udel.edu (S.J.K.); 2 Department of Veterinary and Biomedical Sciences, University of Minnesota, Saint Paul, MN 55108, USA; tjj@umn.edu (T.J.J.); byoumans@umn.edu (B.W.W.)

**Keywords:** microbiome, avian, respiratory, chicken, virome, bioinformatics

## Abstract

Elucidating the complex microbial interactions in biological environments requires the identification and characterization of not only the bacterial component but also the eukaryotic viruses, bacteriophage, and fungi. In a proof of concept experiment, next generation sequencing approaches, accompanied by the development of novel computational and bioinformatics tools, were utilized to examine the evolution of the microbial ecology of the avian trachea during the growth of a healthy commercial broiler flock. The flock was sampled weekly, beginning at placement and concluding at 49 days, the day before processing. Metagenomic sequencing of DNA and RNA was utilized to examine the bacteria, virus, bacteriophage, and fungal components during flock growth. The utility of using a metagenomic approach to study the avian respiratory virome was confirmed by detecting the dysbiosis in the avian respiratory virome of broiler chickens diagnosed with infection with infectious laryngotracheitis virus. This study provides the first comprehensive analysis of the ecology of the avian respiratory microbiome and demonstrates the feasibility for the use of this approach in future investigations of avian respiratory diseases.

## 1. Introduction

Microbiomes are complex environments consisting of eukaryotic viruses, bacteria, archaea, bacteriophage, fungi, and protozoa; and microbiomes exist throughout the body in the oral cavity, intestinal tract, respiratory tract, vaginal tract and skin of both animals and humans [1,2]. The composition within these different microbiome environments varies as the microbial communities participate in unique biological functions. These microorganisms often interact in symbiosis, in which the host organism and the microbiota interact to maintain homeostasis of the host environment [3]. In the bacterial microbiome these functions can include housekeeping functions necessary for microbial life, processes specific to the body-site, and specialized functions for each habitat [4]. Mutualistic symbiotes within the viral microbiome, or virome, have been found to benefit the host by altering innate immunity to other pathogens, both viral and bacterial. For example, gammaherpesvirus 68, was recently found to increase resistance to the bacterial pathogens *Listeria monocytogenes* and *Yersinia pestis* in mice when latent [5,6]. Viruses can also affect infection by other viral pathogens. This can occur due to interference, a phenomenon by which a viral infection causes a cell to be temporarily resistant to infection by other viruses. This type of behavior was observed in a study by Grivel et al. in 2001, in which they found that persistent infection by human herpesvirus 6 could inhibit HIV-1 infection and progression in lymphoid tissue [7]. On the other hand, interactions between viruses such as infectious laryngotracheitis virus, infectious bronchitis virus and New Castle disease virus can lead to a dysbiosis of the avian respiratory microbiome, leading to respiratory disease [8]. Evidence of symbiotic fungi and yeast have also been identified. *Saccharomyces boulardii*, for example, has been found to exhibit probiotic behaviors against pathogens such as *Escherichia coli*, *Vibrio cholera*, *Salmonella* [9] and *Clostridium difficile* in humans [10] and several animals, including turkeys [11].

Recent studies attempting to characterize the respiratory microbiome of poultry have focused primarily on bacteria, as there are well established and rapid methods of sequencing and analyzing this component [12,13,14,15,16,17]. The 16S rRNA gene is commonly used to identify and compare bacteria present in a given sample [18]. Accessible bacterial databases, such as Greengenes [19] and Silva [20], in addition to well-developed bioinformatics pipelines are available to facilitate these analyses [21,22,23]. Glendinning et al. [15] utilized these 16S rRNA gene amplification approaches to characterize the buccal, nasal and lung microbiota of chickens. Utilizing similar methods, Shabbir et al. [17] determined that the lower respiratory tract of healthy flocks of chickens from different farms in Pakistan exhibited high levels of diversity in their microbiota. More recently, Johnson et al. [16] presented a comprehensive analysis of the core bacterial microbiota in the broiler gastrointestinal, respiratory, and barn environments. Although *Lactobacillaceae* were the predominant bacteria found in the trachea, similar to the ileum, the dominant *Lactobacillus* species differed in relative abundance when tracheal and ileum tissues were compared.

Although the bacterial component provides valuable information about the respiratory microbiome, a comprehensive analysis of the avian respiratory microbiota has not been reported. Unlike bacteria, viruses lack a marker gene that can be sequenced and employed for taxonomic classification due to their high genetic heterogeneity [24]. With the advancement of next generation metagenomic sequencing technologies, virome characterization is also possible. Although this method has been successful in identifying viruses [25,26], robust computational methods for analysis of viral metagenomic data are incomplete. The lack of comprehensive bioinformatics pipelines and viral genome databases limit efforts to characterize the virome. Given this limitation and the lack of a comprehensive microbial environment for the broiler chicken, we developed and employed a bioinformatics pipeline and bacteriophage, fungal, and avian viral genome databases to examine a healthy flock of chickens throughout their grow out cycle. These methods were used to detect and quantify eukaryotic DNA and RNA viruses, bacteria, bacteriophage, and fungi. This study provides the first comprehensive analysis of the ecology of the avian respiratory microbiome.

## 2. Materials and Methods

### 2.1. Sample Collection

A 4000 bird antibiotic-free commercial poultry flock, grown in the Jones Hamilton Environmental Research House at the University of Delaware Elbert N. and Ann V. Carvel Research and Education Center, was utilized for this longitudinal study of the broiler respiratory microbiome. The flock was vaccinated in ovo with a recombinant HVT/NDV vaccine and at hatch with a coccidiosis vaccine (5 strains) and a multi-component infectious bronchitis vaccine (Ark, Conn, Delaware type). Birds were grown in the fall on used litter and sampled weekly, beginning at placement, and concluding at 49 days, the day before processing. The flock had no disease or health issues during grow out. Production parameters and health were normal, with total mortality after the first week of 1.4%, average final body weight of 7.95 lb, and a feed conversion ratio of 1.76. Ammonia levels in the house were maintained below 20 ppm. Birds displayed no clinical signs of disease during grow out and veterinary services (necropsy, serology) were not performed. Tracheal swabs were collected at placement and at weekly intervals through day 49 (8 time points). At each time point two samples containing six individual swabs were collected in 3 mL of buffer PV1 (Qiagen, Germantown, MD, USA), frozen immediately on dry ice and stored at −80 °C until use.

Tracheal swabs were also collected and pooled from three respiratory clinical samples submitted to the University of Delaware Poultry Health System (UDPHS) Lasher Laboratory at the University of Delaware. The clinical diagnosis of all three samples was infectious laryngotracheitis (ILT), or ILT complicated by respiratory disease complex (RDC). All three flocks tested positive for infectious bronchitis virus (IBV) and ILTV by PCR, and negative for avian influenza virus (AIV). Tracheal swabs were collected in BHI broth and frozen immediately at −80 °C.

### 2.2. Nucleic Acid Extraction and Sequencing

After thawing on ice, the pooled samples were gently homogenized, split into two tubes and then centrifuged (7000× *g*; 5 min; 4 °C) to form pellets. Preliminary experiments demonstrated insufficient genetic material in the resulting supernatant for library construction. Total RNA was isolated from one pellet using the Qiagen (previously MoBio) Viral Nucleic Acid Extraction Kit following the manufacturer’s protocol. DNA was isolated from the duplicate pellet using the Qiagen Blood and Tissue Kit following the manufacturer’s protocol. Both DNA and RNA sequencing was performed for each time point using the Illumina HiSeq platform producing 1 × 100 nucleotide single-end reads by the University of Delaware Sequencing Core Facility. 

### 2.3. 16S rRNA Amplicon Sequencing and Analysis

The V4 hypervariable region of the bacterial 16S rRNA gene was extracted and amplified using PCR with primers 515F (‘5- GTGCCAGCMGCCGCGGTAA-3′) and 806R (‘5-GGACTACHVGGGTWTCTAAT-3′), as previously described [16,27]. The conditions of the first PCR reaction used were an initial denaturation step at 95 °C for 5 min, followed by 25 cycles of 98 °C for 20 s, 55 °C for 15 s, and 72 °C for 1 min, with a final extension at 72 °C for 5 min. The product was diluted 1:100 and used in a second PCR reaction. The second PCR reaction consisted of an initial denaturation step at 95 °C for 5 min, followed by 10 cycles of 98 °C for 20 sec, 55 °C for 15 sec, and 72 °C for 1 min, with a final extension at 72 °C for 5 min. The pooled, size-selected sample was denatured with 0.2N NaOH, diluted to 8 pM in Illumina’s HT1 buffer, spiked with 20% PhiX, and heat denatured at 96 °C for 2 min immediately prior to loading. The amplicons were sequenced at the University of Minnesota Genomics Center (Minneapolis, MN, USA) using an Illumina MiSeq 600 cycle v3 kit.

Following sequencing, samples were sorted by barcode to generate individual fastq files. Each sample was assessed for quality and assembled into contigs using PEAR’s default parameters, with the modification that the quality score threshold was set to 30. Samples were further filtered and analyzed using Mothur version 1.35.1 [23] and MiSeq SOP [28]. OTUs were generated using 97% sequence similarity. Mothur’s implementation of the SILVA database (v123) was used for classification of OTUs. Alpha-diversity was measured using the Shannon diversity index [29,30]. Relative abundance, mean relative abundance and genera frequency were also calculated. These data were represented by pie charts, phylogenetic trees and networks using the R library GraPhlAn and Cytoscape [31,32]. A Pearson correlation matrix between bacteria and bacteriophage was constructed using the R library Corrplot [33].

### 2.4. Eukaryotic Virus, Bacteriophage and Fungal Analysis

Raw DNA-Seq and RNA-Seq reads were processed using BiomeSeq [34]. Individual sequence files were first analyzed for per-base sequence quality, per sequence quality, sequence length distribution, duplicate sequences, and overrepresented Kmers. Reads with a quality phred score below 30, reads under 100 nucleotides and adapter sequences were removed. The remaining reads were then aligned to the reference host genome (Gallus *gallus* Annotation Release 104) using Bowtie2 alignment algorithm [35,36]. Only unmapped reads were extracted and analyzed further. This step removes host genome contamination from the data, increasing analytical efficiency [37]. Determining the amount of host genome sequence in the library is also required for quantification. The remaining reads were then aligned to microbial databases including a bacteriophage, a fungal and an avian-specific viral genome database. 

The avian-specific viral genome database contains full genome reference sequences of both DNA and RNA avian viruses obtained from the National Center for Biotechnology Information (NCBI) reference sequences. The avian DNA viral database contains 48 viral elements from 9 unique families and the avian RNA viral database contains 77 viral elements from 13 families (Table 1). The avian DNA and RNA viral databases are organized by the classification of their viral structure and genome organization and are available upon authorization at https://sites.udel.edu/aviangenomics (accessed on 30 March 2021). DNA viruses are organized hierarchically by whether the virus is double- or single-stranded and whether the virus is enveloped or non-enveloped. RNA viruses are organized hierarchically by whether the virus is double- or single-stranded, negative or positive sense, segmented or non-segmented and whether the virus is enveloped or non-enveloped.

The bacteriophage database consists of complete bacteriophage sequences from the NCBI Reference Sequence databases. The bacteriophage database contains 2212 complete genomes and is organized hierarchically by taxonomic resolution including order, family, genera and species. The fungal database consists of complete fungal sequences from the NCBI Reference Sequence databases. The database contains 1281 complete fungi genomes and is organized hierarchically by taxonomic resolution including phyla, class, order, family, genera and species.

A sequence similarity-dependent approach for detecting microbes, such as this, contributes to the rapid detection of known microbes while also allowing for the quantification of biodiversity which similarity-independent approaches lack [38]. For each individual sample, the reads that mapped to each microbe were normalized based on the genome length of both microbe and reference per 100,000 host cells using the following equation [39]:

(1) $$microbial\;abundance=\;\frac{2\;\times \;\frac{number\;of\;reads\;mapped\;to\;microbial\;genome}{microbe\;genome\;size}}{\frac{number\;of\;reads\;mapped\;to\;chicken\;genome}{chicken\;genome\;size}}\times \;10^{5}$$

Relative microbial abundance, mean relative abundance and species frequency were also calculated. These data were represented by pie charts, phylogenetic trees and networks using the R library GraPhlAn and Cytoscape [31,32]. Alpha diversity was measured using Shannon diversity index [29,30]. In addition, stacked bar plots and heatmaps were generated with the R library PhyTools [40]. A Pearson correlation matrix between bacteria and bacteriophage was constructed using the R library Corrplot [32].

## 3. Results

### 3.1. Avian Respiratory Eukaryotic Viral Diversity

As described in Materials and Methods, a healthy antibiotic-free commercial poultry flock, grown at the University of Delaware Elbert N. and Ann V. Carvel Research and Education Center, was utilized for this longitudinal study of the broiler respiratory microbiome. Tracheal swabs were collected at placement and at weekly intervals through day 49 (8 time points). Tracheal swabs from 12 birds were collected at each time point as two pools of six swabs. The two pools were combined and used for the extraction of DNA and RNA. DNA-Seq and RNA-Seq libraries were constructed and sequenced and the V4 hypervariable region of the 16S rRNA gene was also amplified and sequenced. A total of 339,319,712 trimmed DNA-Seq reads and 440,442,599 trimmed RNA-Seq reads were generated from 16 libraries. A total of 78.787 giga base pairs (Gbp) of high-quality nucleotide sequences were obtained (Table S1). An average of 88% of the DNA reads mapped to the chicken genome, while an average of 53% of RNA reads mapped to the chicken genome. 

Unmapped DNA and RNA reads from the eight weekly broiler respiratory samples, each representing a pool of 12 birds, were aligned to an avian specific viral genome database consisting of 77 complete avian RNA viral genomes and 48 complete avian DNA viral genomes (Table 1). The 5163 reads which aligned to the avian viral DNA database and the 71,936 reads which aligned to the avian viral RNA database were analyzed as described in the Materials and Methods.

**Figure 1:**
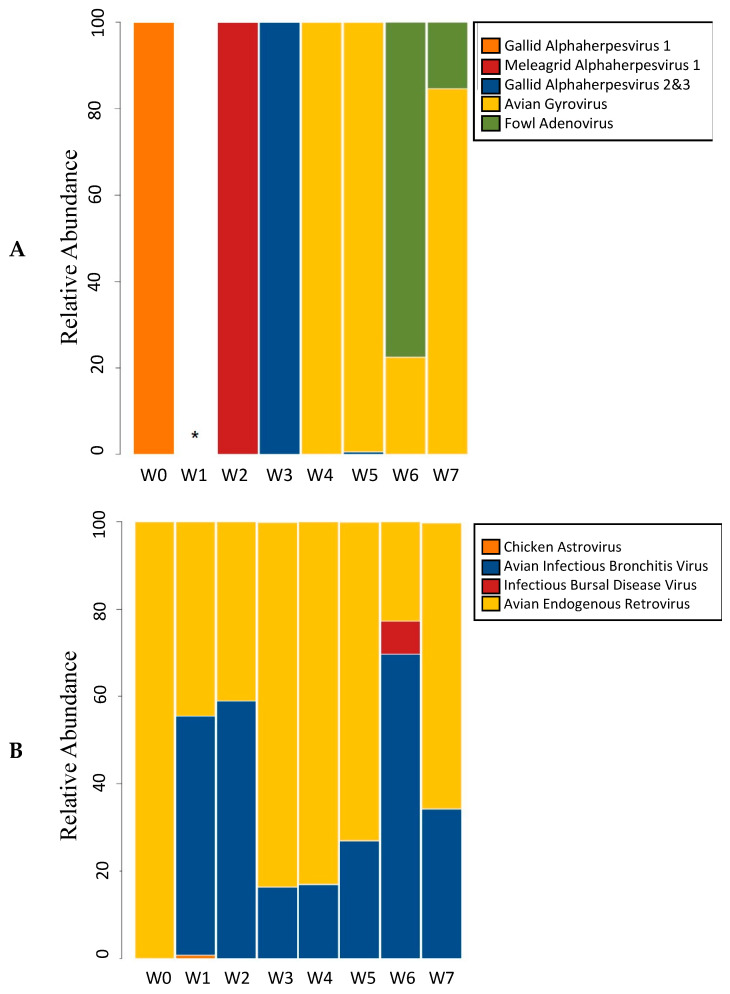
Normalized relative abundance of detected DNA (**A**) and RNA (**B**) viral species at each time point. * No DNA viruses detected at Week 1.

**Figure 2:**
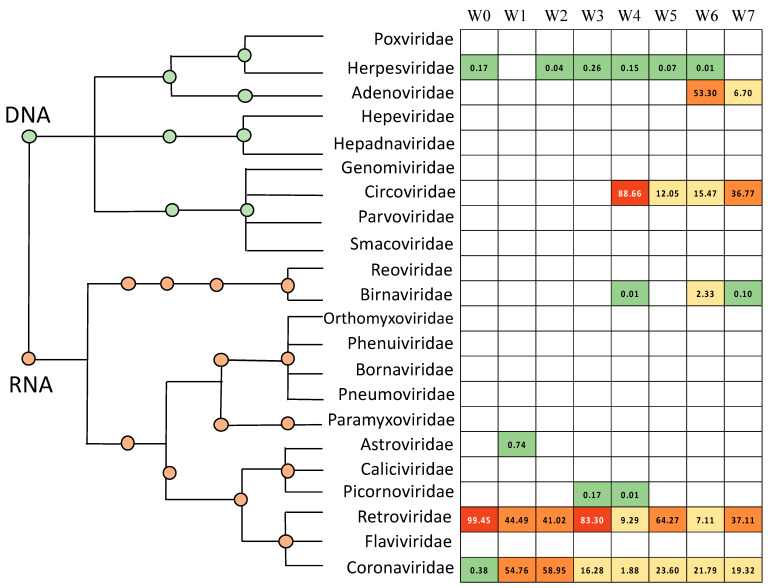
Heat map with phylogenetic tree representing the detection frequency of eukaryotic viral families at each individual week. Color corresponds to the range of relative abundance of each week from 0 to 100%. The sum of each column, or week, is 100%.

**Figure 3:**
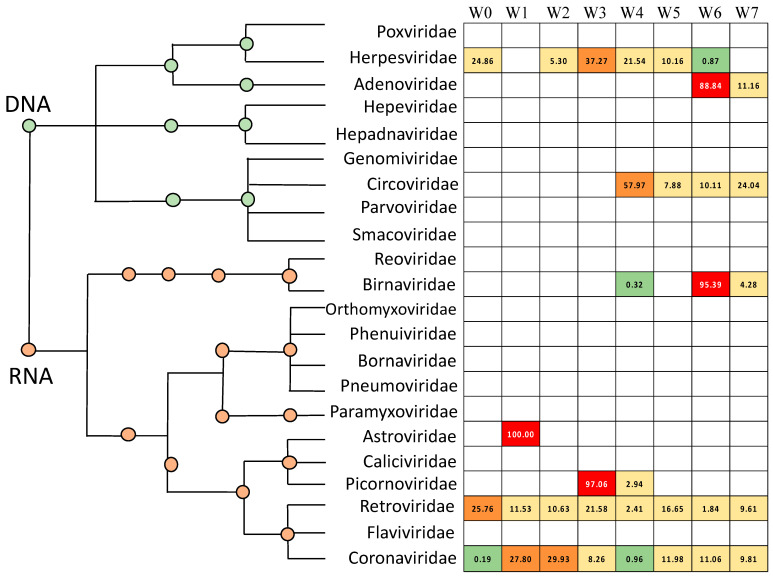
Heat map with phylogenetic tree representing the detection intensity of each eukaryotic viral family from hatching to processing. Color intensity corresponds to the range of relative abundance of each family from 0 to 100%. The sum of each row, or viral family, is 100%.

A total of 11 viral species, representing 9 genera and 8 families, were identified from the avian respiratory tract during the seven week grow out period (Table S2). Normalized viral abundance was calculated for each eukaryotic viral species for each week (Figure 1, Table S3). At placement, or week 0, *Gallid herpesvirus 1* was the only DNA virus detected (Table S3). Relatively small amounts of *Meleagrid herpesvirus 1* were detected at week 2, and *Gallid herpesvirus 2 and 3* were detected in small quantities from weeks 3–6. Two other viral DNA families were detected later during growth. Circoviridae (avian gyrovirus) first appears in the avian respiratory tract at four weeks of age, while avian adenovirus was initially detected at week 6. The relative abundance of the DNA viruses of the respiratory tract can be examined on the basis of their percent relative abundance to the other viruses in each sample (Figure 2, Table S4) or with respect to the relative distribution of the specific virus families throughout the 7 week period (Figure 3, Table S5). It is notable that when Circoviridae and Adenoviridae first appear (Week 4 and Week 6, respectively) they represent the highest viral abundance in the tracheal sample (88.66% and 53.30%, respectively, Figure 2). They also represent the highest relative abundance of these virus families observed during flock growth (57.97% and 88.84%, respectively, Figure 3).

Five eukaryotic RNA virus families were identified in the tracheal samples during grow out. Since only representative reference sequences were used to construct the avian eukaryotic virus database, the genome alignment bioinformatics approach was not able to determine strain or serotype information. The pattern of RNA virus detection differed markedly from the observed patterns of DNA virus detection (Figure 2 and Figure 3). As expected, transcripts from endogenous avian retroelements were detected throughout the growth of the flock. Coronaviridae sequences were also observed in all of the respiratory samples, and they were the most abundant virus family found at Week 1 and Week 2 (Figure 2). Three other RNA virus families were observed in the broiler trachea. Astroviridae and Picornoviridae were observed transiently and in low numbers during Week1 and Weeks 3–4, respectively. In addition, relatively low levels of Birnaviridae, infectious bursal disease virus, were observed in tracheal samples from Weeks 4, 6, and 7. 

**Figure 4:**
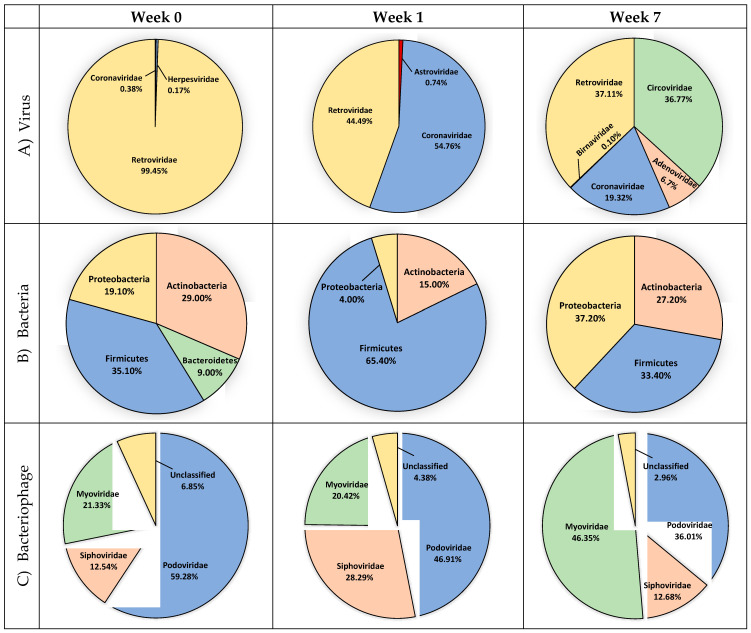

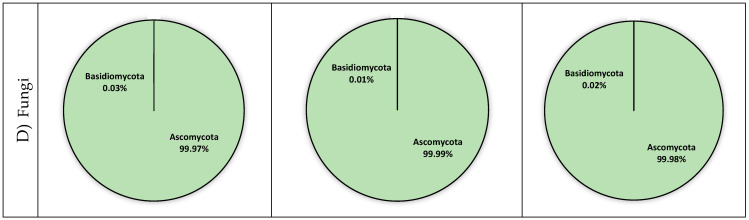
Abundance of (**A**) virus, (**B**) bacteria, (**C**) bacteriophage and (**D**) fungi at Week 0, Week 1 and Week 7. Taxa represented at family (**A**,**C**) and phylum (**B**,**D**) level.

**Figure 5:**
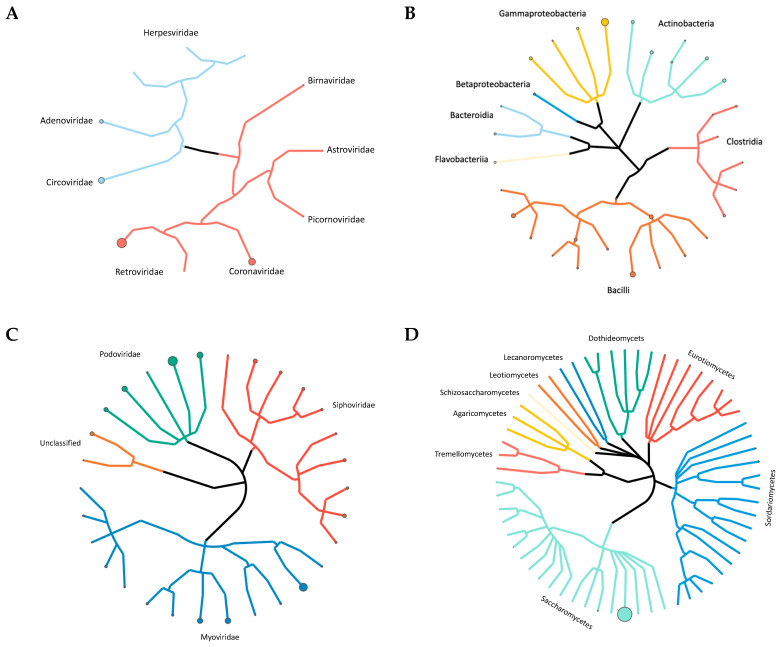
Phylogenetic tree of (**A**) virus, (**B**) bacteria, (**C**) bacteriophage and (**D**) yeast and fungi. Node diameter indicates average abundance at species (**A**,**C**,**D**) and genera (**B**) level. Taxonomic levels range from phylum to genera (**B**), order to species (**C**) phylum to species (**D**). Viruses (**A**) are organized according to structural classification.

Figure 4A compares the avian respiratory viral microbiome of newly placed chickens, correlating with the microbial environment of a commercial hatchery (Week 0), with birds who have spent 1 week on litter (Week 1) to that of mature broiler chickens at the time of processing (Week 7). An examination of the viral microbiome (Figure 4A, Table S4) revealed only Coronaviridae and Herpesviridae at hatch. After 1 week, Coronaviridae are well established in the birds. After 6 more weeks, a more diverse and complex viral environment was observed, where avian adenovirus, chicken anemia virus and infectious bronchitis virus dominate. Average normalized abundance was calculated at the family level (Figure 5A, Table S5). Alpha diversity was also calculated at each week using Shannon Diversity Index (Table 2). Both RNA viruses and DNA viruses exhibited their lowest diversity at placement (Week 0, H = 0.041 and H = 0.000, respectively). DNA viruses saw an increase in diversity at Week 4 and exhibited their highest diversity at Week 7 (H = 0.867). The RNA virus population exhibited the highest diversity at Week 6 (H = 1.480).

### 3.2. Bacterial Diversity

A total of 50,181 reads were obtained from sequencing the V4 hypervariable region of the 16S rRNA gene (Table S1). Week 2 and Week 6 were omitted from analyses due to the low number of processed reads. Processing and analysis was performed on the samples following the protocol discussed in the Materials and Methods. Following processing, a total of 353 operational taxonomic units (OTUs) were obtained.

A total of 24 unique bacterial genera were identified and extended from 4 phyla, 7 classes, 13 orders and 24 families (Table S6). Average abundance was calculated in a similar manner to the previous analyses. The phyla Firmicutes made up most of the bacteria with an average abundance of 56.17%, followed by Proteobacteria (39.28%), Actinobacteria (24.00%) and Bacteroidetes (5.78%). The average relative abundance of all phyla, classes, orders, families, genera and species are available in Table S6. Within the Firmicutes phylum, Bacilli was the most abundant class with an average abundance of 49.02% followed by Clostridia (7.15%). Within the Proteobacteria phylum, Gammaproteobacteria was the most abundant class with an average abundance of 36.28% followed by Betaproteobacteria (3.00%). Actinobacteria (24.00%) was the only class in the Actinobacteria phyla. The Bacteroidia (3.38%) and Flavobacteria (2.40%) classes made up the Bacteroidetes phyla (Figure 5B). 

A comparison of the Week 0 to the Week 1 bacterial microbiome (Figure 4B) reveals that the Bacteroidetes present at hatch (9.00%) are lacking by Week 1 and the Actinobacteria and Proteobacteria are significantly reduced. The Firmicutes nearly double in abundance by Week 1 at the expense of these three families. By the end of the grow out cycle more balanced populations of Proteobacteria (37.20%), Actinobacteria (27.20%) and Firmicutes (33.40%) are observed. Calculations of Alpha diversity (Table 2) showed a consistently diverse bacterial population, which is highest at placement and lowest near the end of the grow out period.

We also investigated the frequency of specific bacterial genera during the grow out cycle (Table S7). Three different population patterns were observed. At placement, several genera from all four phyla are represented. Representing the Actinobacteria are the *Corynebacteriaceae* (6%), *Brevibacterium* (9%), the *Brachybacterium* (8%) and the *Yaniella* (1%). These are observed in lower abundance throughout flock growth. At placement the Proteobacteria are predominantly represented by the *Pseudomonas* (13%) which are not found in significant levels after Week 1. As shown in Figure 4B, Week 0 is the time significant numbers of Bacteroidetes are observed (*Chryseobacterium*, 7% and *Alloprevotella* 2%). The predominant Firmicutes seen at placement, and consistently observed at high levels throughout growth are the *Lactobacilli* (5.1%). 

Once established on litter, the avian bacterial respiratory microbiome is consistent for the first 4 weeks and is dominated by the Lactobacilli, averaging almost 40% of the detected OTUs. Other *Bacillaceae* and *Staphylococcus* from the Firmicutes as well as Actinobacteria phyla are also consistently observed. By Week 7, a significant shift to the Proteobacteria and Actinobacteria occurs in the respiratory tract (Figure 4B). While the relative abundance of *Lactobacilli* drops to 14.8%, significant numbers of Gallibacterium (37%) and Corynebacteriaceae (22%) are now present (Table S7).

### 3.3. Bacteriophage Diversity

The unmapped DNA sequences were also aligned to a bacteriophage database consisting of 3429 complete genome sequences. A total of 504,682 reads aligned to bacteriophage genomes (Table S1). A total of 31 unique bacteriophage species extended from 1 classified and 1 unclassified order, 3 classified and 1 unclassified families, and 8 classified and 4 unclassified genera were identified (Table S8). Normalized abundance, percent relative abundance and average abundance was calculated similar to the previous analyses (Tables S8–S10). Of the classified families of bacteriophage observed, the Myoviridae were the most abundant with an average normalized abundance of 70.99%, followed by Podoviridae (40.19%) and Siphoviridae (31.16%) (Figure 4C, Figure 5C, Table S8). The most abundant species of bacteriophage was *Enterobacteria phage RB55* with an average normalized abundance of 39.16%. 

**Figure 6:**
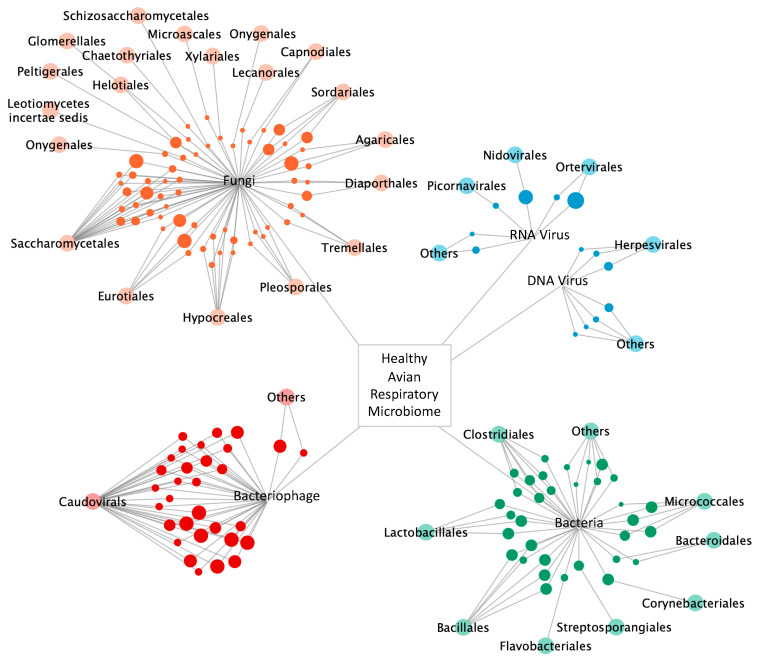
Microbial network of the complete healthy avian respiratory microbiome including detected RNA viruses, DNA viruses, yeast and fungi, bacteria, and bacteriophage. Taxa nodes are arranged by order. Node diameter correlates to taxa frequency.

We also investigated the frequency of specific bacteriophage species observed during the grow out cycle (Figure 6, Table S10). Salmonella phage RE-2010, Enterobacteria phage IME10, Enterobacteria phage T7, Enterobacteria phage VT2phi_272, Escherichia phage TL-2011b, Stx2 converting phage vB_EcoP_24B and Stx2-converting phage 1717 were detected in all eight weeks whereas Salmonella phage SJ46, Shigella phage SfIV, Enterobacteria phage lambda, and Enterobacteria phage YYZ-2008 appeared in seven of the eight weeks. Alpha diversity was calculated at each week using Shannon Diversity Index (Table 2). Samples exhibited the lowest bacteriophage diversity at Week 2 (H = 2.111) and the highest diversity at Week 3 (H = 2.922). When comparing bacteriophage families from hatching to processing, Myoviridae increased by 25.02% while Podoviridae and unclassified bacteriophage decreased by 23.27% and 3.89%, respectively (Figure 4C). 

**Figure 7:**
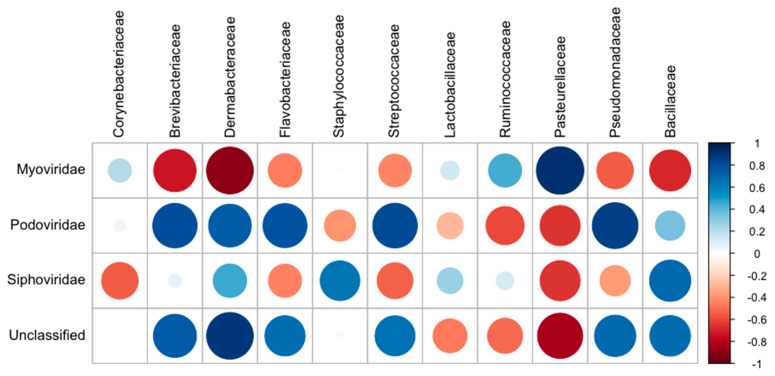
Correlation matrix comparing bacteria and bacteriophage taxa at the family level. Node diameter corresponds to level of correlation. Node color corresponds to the Pearson correlation coefficient and ranges from -1 to 1 indicated by red and blue, respectively.

Correlations between detected bacteria and bacteriophage were analyzed based on the Pearson coefficient of correlation. A total of 44 correlations were calculated between 4 families of bacteriophage and 11 families of bacteria (Figure 7). Strong, positive correlations were observed between the bacteriophage families and bacterial families present in the trachea. Siphoviridae (R = 0.459), Podoviridae (R = 0.743) and the unclassified bacteriophage (R = 0.887) exhibited strong positive correlations with the Dermabacteraceae. Podoviridae showed positive correlations with the Brevibacteriaceae (R = 0.802), the Pseudomonadaceae (R = 0853), Flavobacteriaceae (R = 0.788) and Streptococcaceae (0.812) which are found in the avian trachea at hatch. Siphoviridae had a positive correlation with the Staphylococcaceae (R = 0.641), which are found in the trachea throughout growth.

### 3.4. Fungal Diversity

The unmapped DNA sequences were also aligned to a fungi database consisting of 1281 genomes. A total of 1964 reads aligned to fungi genomes (Table S1). A total of 61 unique fungal species were identified which extended from 2 phyla, 9 classes, 20 orders, 37 families and 50 genera (Table S11). Normalized abundance, percent relative abundance and average abundance was calculated similar to the previous analyses (Tables S11–S13). Of the 2 Phyla, Ascomycota was by far the most abundant. The average abundance of all phyla, classes, orders, families, genera and species are available in Table S11. Within the Ascomycota phylum, the most abundant class was Saccharomycetes with an average abundance of 98.76%. Within the Basidiomycota phylum, the most abundant classes were Agaricomycetes with an average abundance of 0.05% and Tremellomycetes (0.03%). 

We also investigated the frequency of specific fungi species observed during the grow out cycle (Figure 6, Table S13). *Laccaria bicolor*, *Penicillium chrysogenum* and *Wickerhamomyces ciferrii* were detected in all eight weeks whereas *Tetrapisispora phaffii* and *Aspergillus oryzae* appeared in seven of the eight samples. Twenty-six of the sixty-one fungal species were only detected in one sample. We also observed no shifts in fungal microbial communities during the experiment (Figure 4D, Figure 5D). Alpha diversity was calculated at each week using Shannon Diversity Index (Table 2). The dominance of a single fungal species resulted in low levels of Alpha diversity at each week.

### 3.5. Poultry with Respiratory Disease Exhibit a Dysbiosis of the Respiratory Virome

**Figure 8:**
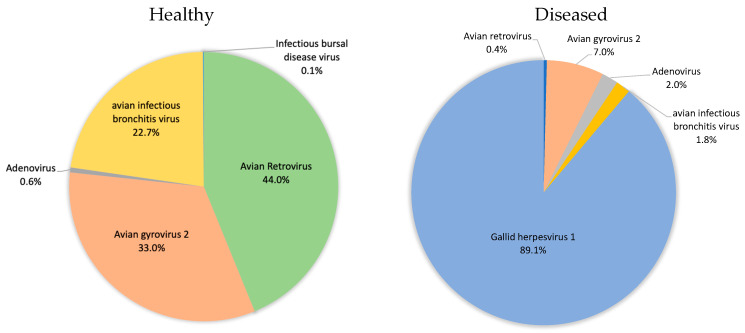
Changes in the eukaryotic viral microbiome associated with avian respiratory disease.

The utility of using a metagenomic approach to study the avian respiratory virome was evaluated by examining birds diagnosed as being infected with infectious laryngotracheitis virus, an avian alphaherpesvirus. Tracheal samples from three birds were pooled, nucleic acids were extracted, and sequencing libraries were constructed, sequenced, and analyzed using BiomeSeq as described earlier. 32,478 reads were aligned to the avian virome database (Table 3). As shown in Figure 8, the relative composition of the virome underwent a shift in these diseased birds. The normal healthy virome consisting predominantly of avian infectious bronchitis virus and avian gyrovirus went from 55.7% to 8.8% of the relative virus population. Infectious laryngotracheitis virus, undetected in the trachea of healthy birds represented 89.1% of the eukaryotic virus population in the trachea of the diseased birds, confirming the clinical diagnosis.

### 3.6. The Avian Microbiome

The development of next generation sequencing approaches, accompanied by the further development of novel computational and bioinformatics tools enabled us to examine the evolution of the microbial ecology of the avian trachea (eukaryotic virus, bacteria, bacteriophage, and fungi) during the growth of this commercial flock. Figure 6 is a representation of the complex ecology of the respiratory microbiome of the broiler chicken. In this microbial network, nodes of bacteria, bacteriophage, eukaryotic viruses, and fungi are arranged by order, while the diameter of the node depicts taxa frequency from 1–8 samples.

## 4. Discussion

A detailed characterization of the bacterial microbiota of the commercial broiler chicken was recently published [16]. This study examined the core bacterial microbiota of the broiler gastrointestinal, respiratory, and barn environments. *Lactobacillus* was found to be the dominant bacterial taxon of the trachea, although the trachea was found to also contain *Staphylococcus*, *Streptococcus*, *Ruminococcus*, and *Xanthomonas.* This study by Johnson et al. [16] was conducted as a longitudinal study from Day 7 to Day 42 and utilized multiple flocks so that microbiome composition could be correlated with performance. 

The goal of our study was to determine the feasibility of expanding the characterization of the broiler respiratory microbiome beyond the bacterial component. Although emphasizing the eukaryotic virome, another objective was to use next generation sequence data to determine the bacteriophage and fungal composition of the avian respiratory tract. As expected, the majority of the sequences obtained were generated from chicken RNA or DNA. In a similar study [39], 95% of reads generated from sequencing human blood successfully mapped to a human reference genome. Only 0.1% of the reads mapped to eukaryotic viral sequences. However, the power of utilizing a next generation sequencing approach enabled the authors to identify human adenovirus at a median of 1 sequencing read per individual. In our study, pooling samples only allows us to determine and quantify the presence or absence of specific microbial components in the population. Now that the power and utility of this approach has been confirmed, we are currently extending our microbiome analysis to individual birds within a flock, enabling the determination of prevalence.

This required the development of a unique bioinformatics tool (BiomeSeq) that utilizes a sequence-dependent approach [34]. To determine and quantify the relative abundance of these microbial elements RNA-Seq and DNA-Seq derived sequences were initially aligned to the avian genome, followed by aligning the remaining sequences to avian-virus specific databases, a bacteriophage database, and a fungus database. Alignment to a host genome sequence followed by alignment to specific microbial databases allows the microbial community in a given sample to be quantified, a unique property of this sequence-dependent bioinformatics approach. This does not preclude the use of the same data in a sequence-independent manner, allowing for a more traditional metagenomics approach that can be used to create contigs that could then be utilized to identify and sequence novel viral elements. Using either method, functional genes and metabolic pathways can be identified using BLASTP and KEGG databases [41].

Previous avian virome studies have focused on the RNA virus community of the avian gut [42,43]. Tracheal swabs of the avian respiratory tract are not amenable to traditional viral enrichment strategies such as centrifugation or filtration because of their small volume, the relatively low viral concentrations in the samples, and the nuclease rich environment (unpublished data). Although we have successfully determined the composition of the avian respiratory microbiome from pools of two tracheal swabs, for this proof of concept study we pooled twelve swabs in two samples to increase viral RNA yield. Swab material is collected in a chaotropic buffer that was rapidly frozen in order to preserve the integrity of viral RNA. In addition, the decision to utilize a sequence-dependent approach to analyze the sequence data necessitated the development of specific databases. For the eukaryotic virome, an avian virus-specific whole genome database was developed (Table 1). Representative whole genomes from 22 viral families (9 DNA viruses, 13 RNA viruses) are represented in the database. Once chicken genome sequences are removed from the RNA-Seq and DNA-Seq library fastq files, alignment to the avian-specific viral database and subsequent analysis is rapid and efficient.

An examination of the avian respiratory viral microbiome confirmed the presence of a dynamic and diverse community. The commercial broiler flock utilized in this study was vaccinated in ovo with a live Marek’s disease virus vaccine (SB-1) and a live recombinant herpesvirus of turkeys (HVT) vaccine expressing Newcastle disease virus genes. At hatch, chicks were also vaccinated by spray with a multivalent infectious bronchitis virus (avian coronavirus) vaccine before placement (Mass and Ark). The consistent presence of herpesviruses and coronaviruses in the respiratory tract is consistent with vaccination with these two live vaccines, coupled with the expected presence of these avian viruses in the environment. As our avian eukaryotic virus database contains a limited number of elements we were unable to serotype the infectious bronchitis or infectious bursal disease virus strains identified in the healthy flock. Efforts are underway to determine if such identifications can be made. In cases such as in flocks acutely infected with a respiratory viral pathogen, where large number of strain- or species-specific sequencing reads are generated, we have been able to generate de novo the complete sequence of the pathogen involved (manuscript in preparation). 

As predicted, as the birds aged, the complexity and diversity of the viral community also increased. Of particular note are the appearance of infectious bursal disease virus (Birnaviridae) at Week 6 and chicken anemia virus (Circoviridae) at Week 4. Broiler breeders are vaccinated in order to maximize the amount of maternal antibodies to these potential pathogens in the newly hatched chick. By Week 4 maternal antibody levels should be reduced to the level where colonization of the respiratory tract by these viruses is likely. However, by Week 4, the avian adaptive immune system has matured. Consequently, the initially observed relative abundance of these viruses is the highest level observed (57.97% of the detected chicken anemia virus sequences, Figure 3). The rapid reduction in the amount of these viruses in the respiratory tract is most likely due to the activation of the avian adaptive immune system. A similar observation is seen with the appearance in Week 6 of avian adenovirus in the avian respiratory tract. Avian adenoviruses are commonly isolated from the avian respiratory tract. Finally, picornaviruses and astroviruses are commonly found in the digestive tract of chickens [39], not the respiratory tract. However, it is not surprising that representatives from these virus families would be transiently observed in the respiratory tract during their initial colonization of the bird.

Once the temporal development of the healthy avian respiratory microbiome had been determined, efforts were made to compare the respiratory virome of healthy birds to the virome of birds diagnosed with respiratory disease. Through the auspices of the UDPHS we were able to obtain tracheal swabs from submissions to the University of Delaware poultry diagnostic laboratory. These birds were clinically diagnosed with infectious laryngotracheitis and this diagnosis had been confirmed through molecular testing (PCR). The respiratory virome of these diseased birds was found to be dominated by infectious laryngotracheitis virus (89.1%). ILTV was not identified in the healthy flock at any time during growth of the flock. The displacement of the normal healthy avian respiratory virome by ILTV was consistent with the diagnosis and confirmed the ability of the metagenomic method to detect changes in avian respiratory microbiome composition. 

Consistent with the observations of Johnson et al. [16] we observed that the bacterial microbiome of the avian respiratory tract was dominated by the Lactobacilli. The bacterial microbiome of the newly hatched chick was more complex than expected. Although *Lactobacilli* (6.1%) were the dominant Firmicutes, Bacteroidetes (*Chryseobacterium*, 7%), Proteobacteria (*Pseudomonas*, 13%), and Actinobacteria (*Brevibacterium*, 9%) were also observed in significant numbers. The majority of the bacteriophage found in the avian respiratory tract was *Enterobacteria phage RB55* of the Myoviridae family. The presence of this bacteriophage correlated with *Gallibacterium* (Pasteurellaceae), an abundant bacterial species found in the last two weeks of growth. Interactions between bacteriophage and bacteria are known to have a significant impact on host health (24). Bacteriophages may also help control bacterial populations, influencing bacterial diversity and contributing to the dysbiosis of the respiratory microbiota during disease. Little is known about the diversity and role of fungi in the respiratory tract of the avian and further studies are needed to determine the relevance of the high normalized relative abundance of the Basidiomycota observed in this flock.

This preliminary study will be confirmed and expanded by examining the respiratory microbiome of multiple flocks from multiple companies, by examining multiple grow-out cycles from the same flock in order to determine seasonal effects and flock consistency, and by examining flocks grown under different production systems (antibiotic free, organic, free range and traditional). These results could also be compared with multi-age backyard flocks from the same geographic area. 

## 5. Conclusions

Ecological niches such as the avian respiratory tract are complex microbial environments consisting of eukaryotic viruses, bacteria, archaea, bacteriophage, fungi, and protozoa, all of which contribute to its microbial ecology. The introduction of an infectious agent can disrupt this environment, resulting in disease. Most microbiome studies have focused primarily on bacteria, as there are well established and rapid methods of sequencing and analyzing conserved ribosomal genes. With the advancement of next generation metagenomic sequencing technologies, the characterization of other microbiome components is possible. We developed and employed a bioinformatics pipeline to examine a healthy flock of chickens throughout their grow out cycle. This report utilized a longitudinal study of one commercial antibiotic-free broiler flock to develop the tools needed to conduct a comprehensive analysis of the microbial ecology of the avian respiratory tract (Figure 6). These methods were used to detect and quantify eukaryotic DNA and RNA viruses, bacteria, bacteriophage, and fungi, and provide the first comprehensive analysis of the ecology of the avian respiratory microbiome. It was also used to demonstrate the dysbiosis exhibited in the respiratory virome of birds diagnosed with infectious laryngotracheitis.

## Figures and Tables

**Table 1:** Avian specific viral genome database structure.

Database	Classification				Virus Family	Complete Genomes
Avian DNA Viral Database	Double/Single Stranded ^a^			Enveloped ^d^	Hepeviridae	1
					Hepadnaviridae	1
	Single Stranded			Non- Enveloped	Genomoviridae	3
					Parvoviridae	7
					Circoviridae	10
					Smacoviridae	3
	Double Stranded			Enveloped	Poxviridae	3
					Herpesviridae	6
	Double Stranded			Non- Enveloped	Adenoviridae	14
Avian RNA Viral Database	Double Stranded		Segmented ^c^	Non- Enveloped	Reoviridae	5
					Birnaviridae	1
	Single Stranded	Positive ^b^	Non- Segmented	Enveloped	Retroviridae	5
					Flaviviridae	3
					Coronaviridae	5
	Single Stranded	Positive	Non- Segmented	Non- Enveloped	Astroviridae	5
					Caliciviridae	1
					Picornaviridae	17
	Single Stranded	Negative	Segmented	Enveloped	Orthomyxoviridae	16
					Phenuiviridae	1
					Bornaviridae	3
					Pneumoviridae	1
	Single Stranded	Negative	Non- Segmented	Enveloped	Paramyxoviridae	14

^a^ single stranded, double stranded or single/double stranded DNA and RNA viruses. ^b^ positive-sense or negative-sense RNA viruses. ^c^ segmented or non-segmented RNA viruses. ^d^ enveloped or non-enveloped DNA and RNA viruses.

**Table 2:** Shannon diversity of respiratory microbes in a healthy broiler flock over time.—no data.

Time	RNA Virus	DNA Virus	Bacteria	Phage	Fungi
Placement	0.041	0.000	2.707	2.218	0.022
Week 1	1.290	0.000	2.468	2.531	0.286
Week 2	1.108	0.000		2.111	0.096
Week 3	0.722	0.000	2.251	2.922	0.165
Week 4	0.738	0.013	2.499	2.756	0.151
Week 5	0.910	0.035	1.925	2.294	0.026
Week 6	1.480	0.534		2.134	0.013
Week 7	1.092	0.867	1.935	2.087	0.134

**Table 3:** Sequencing data generated by DNA-Seq and RNA-Seq for samples from diseased birds.

Sample	Trimmed for Quality	Map to Host	Not Mapped to Host	Map to Virus DB
DNA	23,547,613	21,003,097	2,544,516	32,478
RNA	8,680,175	8,217,793	462,382	

## Data Availability

The data presented in this study are available on request from the corresponding author at https://sites.udel.edu/aviangenomics (accessed on 30 March 2021).

## Supplementary Materials

The following are available online at https://www.mdpi.com/article/10.3390/microorganisms9040721/s1, Table S1: Sequencing data generated by DNA-Seq, RNA-Seq, and 16S-rRNA. Table S2: Average normalized relative abundance of viruses detected. Table S3: Normalized abundance of detected eukaryotic viruses at the species level. Table S4: Normalized percent relative abundance of detected eukaryotic viruses at the species level. Table S5: Normalized percent relative abundance of detected eukaryotic viruses at the family level. Table S6: Average relative abundance of detected bacteria. Table S7: Relative abundance of detected bacteria at the genera level. (Taxa that could not be assigned a genus are displayed using the highest taxonomic level that could be assigned to them: * (family), ** (class), or *** (order)). Table S8: Average normalized relative abundance of detected bacteriophage. Table S9: Normalized abundance of detected bacteriophage at the species level. Table S10: Normalized percent relative abundance of detected bacteriophage at the species level. Table S11: Average normalized relative abundance of detected fungi. Table S12: Normalized abundance of detected fungi at the species level. Table S13: Normalized percent relative abundance of detected fungi at the species level.
