# Supplementary material for: Metagenomic Analysis of the Respiratory Microbiome of a Broiler Flock from Hatching to Processing
Source: Microorganisms. 2021 Mar 31;9(4):721. doi: 10.3390/microorganisms9040721 (PMC8065701; doi:10.3390/microorganisms9040721)
Supplement: Supplementary file 1 [file microorganisms-09-00721-s001.pdf]

Table S1. Sequencing data generated by DNA-Seq, RNA-Seq and 16S rRNA

**DNA-Seq**	**Total Reads**	**After Trimming**	**% Mapped Host**	**Mapped to DNA Viral Reads**	**Mapped to Bacteriophage**	**Mapped to Fungi**
Week 0	46,586,608	46,568,465	86.43	1	68,174	545
Week1	43,940,202	43,911,476	81.11	0	63,256	649
Week 2	33,471,831	33,442,860	89.8	1	61,974	48
Week 3	45,131,953	45,108,639	84.81	2	68,330	313
Week 4	44,621,969	44,600,956	90	387	61,412	109
Week 5	38,630,592	38,590,484	88.99	7	51,011	142
Week 6	45,915,721	45,900,494	89.73	4,634	66,310	82
Week 7	41,215,207	41,196,338	90.18	131	64,215	76
**Total**	339,514,083	339,319,712	701	5,163	504,682	1,964
**Average**	42,439,260	42,414,964	88	645	63,085	246

**RNA-Seq**	**Total Reads**	**After Trimming**	**% Mapped Host**	**Mapped to RNA Viral Reads**
Week 0	62375955	62351619	49.21	416
Week1	54,496,930	54,477,530	44.3	16,569
Week 2	51,426,090	51,415,545	65.54	10,016
Week 3	57,566,851	57,551,710	28.76	977
Week 4	56,334,014	56,321,058	61.62	3,660
Week 5	50,937,821	50,926,463	57.56	4,579
Week 6	53,248,431	53,236,536	59.08	29,476
Week 7	54,173,052	54,162,138	61.44	6,243
**Total**	440,559,144	440,442,599	428	71,936
**Average**	55,069,893	55,055,325	53	8,992

**16S rRNA**	**Total Reads**	**OTUs**
Week 0	8,438	47
Week 1	7,550	79
Week 2	6,737	76
Week 4	9,450	63
Week 5	10,718	50
Week 7	7,288	38
**Total**	50,181	353
**Average**	8,364	59

Table S2. Average normalized relative abundance of viruses detected.

Strand		Segments		Envelope		Family		Genus		Species	
double stranded DNA	7.59%			enveloped DNA	0.09%	Herpesviridae	0.09%	Iltovirus	0.02%	Gallid alphaherpesvirus 1	0.02%
								Mardivirus	0.06%	Meleagrid alphaherpesvirus 1	0.01%
										Gallid alphaherpesvirus 2&3	0.06%
				non-enveloped DNA	7.50%	Adenoviridae	7.50%	Aviadenovirus	7.50%	Fowl aviadenovirus	7.50%
single stranded DNA	19.12%			non-enveloped DNA	19.12%	Circoviridae	19.12%	Gyrovirus	19.12%	Avian gyrovirus	19.12%
single stranded RNA	73.95%	positive, non-segmented	73.64%	non-enveloped RNA	0.77%	Astroviridae	0.74%	Avastrovirus	0.74%	Chicken astrovirus	0.74%
						Picornoviridae	0.02%	Sicinivirus	0.02%	Chicken sicinivirus JSY	0.02%
				enveloped RNA	72.87%	Coronaviridae	24.62%	Gammacoronavirus	24.62%	Avian infectious bronchitis virus	24.62%
						Retroviridae	48.25%	Unclassified Retrovirus	48.25%	Avian Endogenous Retrovirus	48.24%
										Avian carcinoma virus	0.01%
		negative, segmented	0.31%	enveloped RNA	0.31%	Birnaviridae	0.31%	Avibirnavirus	0.31%	Infectious bursal disease virus	0.31%

Table S3. Normalized abundance of detected eukaryotic viruses at the species level.

Species	Week 0	Week 1	Week 2	Week 3	Week 4	Week 5	Week 6	Week 7
Gallid alphaherpesvirus 1	33.52	0.00	0.00	0.00	0.00	0.00	0.00	0.00
Meleagrid alphaherpesvirus 1	0.00	0.00	41.97	0.00	31.40	0.00	0.00	0.00
Gallid alphaherpesvirus 2&3	0.00	0.00	0.00	63.84	1186.57	71.12	57.03	0.00
Avian gyrovirus	0.00	0.00	0.00	0.00	727764.39	12256.84	147165.92	68358.83
Fowl aviadenovirus	0.00	0.00	0.00	0.00	0.00	0.00	507048.92	12450.59
Chicken astrovirus	0.00	1490.69	0.00	0.00	0.00	0.00	0.00	0.00
Chicken sicinivirus JSY	0.00	0.00	0.00	42.11	42.11	0.00	0.00	0.00
Avian carcinoma virus	0.00	0.00	0.00	0.00	0.00	78.52	0.00	78.52
Avian infectious bronchitis virus	74.78	109675.86	67740.67	4048.83	15467.52	23999.29	207253.45	35914.02
Infectious bursal disease virus	0.00	0.00	0.00	0.00	64.88	0.00	22191.95	194.65
Avian Endogenous Retrovirus	19470.62	89108.98	47135.34	20718.00	76251.66	65275.35	67618.14	68898.80

Table S4. Normalized percent relative abundance of detected eukaryotic viruses at the species level. Sum of columns = 100%.

Family	Species	Week 0	Week 1	Week 2	Week 3	Week 4	Week 5	Week 6	Week 7	Frequency
Poxviridae		0.00	0.00	0.00	0.00	0.00	0.00	0.00	0.00	0.0
Herpesviridae	*Gallid alphaherpesvirus 1*	0.17	0.00	0.00	0.00	0.00	0.00	0.00	0.00	12.5
	Meleagrid alphaherpesvirus 1	0.00	0.00	0.04	0.00	0.00	0.00	0.00	0.00	12.5
	Gallid alphaherpesvirus 2&3	0.00	0.00	0.00	0.26	0.14	0.07	0.01	0.00	50.0
Adenoviridae	*Fowl aviadenovirus*	0.00	0.00	0.00	0.00	0.00	0.00	53.30	6.70	25.0
Hepeviridae		0.00	0.00	0.00	0.00	0.00	0.00	0.00	0.00	0.0
Hepadnaviridae		0.00	0.00	0.00	0.00	0.00	0.00	0.00	0.00	0.0
Genomiviridae		0.00	0.00	0.00	0.00	0.00	0.00	0.00	0.00	0.0
Circoviridae	*Avian gyrovirus*	0.00	0.00	0.00	0.00	88.66	12.05	15.47	36.77	50.0
Parvoviridae		0.00	0.00	0.00	0.00	0.00	0.00	0.00	0.00	0.0
Reoviridae		0.00	0.00	0.00	0.00	0.00	0.00	0.00	0.00	0.0
Orthomyxoviridae		0.00	0.00	0.00	0.00	0.00	0.00	0.00	0.00	0.0
Phenumoviridae		0.00	0.00	0.00	0.00	0.00	0.00	0.00	0.00	0.0
Birnaviridae	*Infectious bursal disease virus*	0.00	0.00	0.00	0.00	0.01	0.00	2.33	0.10	37.5
Pneumoviridae		0.00	0.00	0.00	0.00	0.00	0.00	0.00	0.00	0.0
Paramyxoviridae		0.00	0.00	0.00	0.00	0.00	0.00	0.00	0.00	0.0
Astroviridae	*Chicken astrovirus*	0.00	0.74	0.00	0.00	0.00	0.00	0.00	0.00	12.5
Picornoviridae	*Chicken sicinivirus JSY*	0.00	0.00	0.00	0.17	0.01	0.00	0.00	0.00	25.0
Retroviridae	*Avian Endogenous Retrovirus*	99.45	44.49	41.02	83.30	9.29	64.20	7.11	37.06	100.0
	Avian carcinoma virus	0.00	0.00	0.00	0.00	0.00	0.08	0.00	0.04	25.0
Flaviviridae		0.00	0.00	0.00	0.00	0.00	0.00	0.00	0.00	0.0
Coronaviridae	*Avian infectious bronchitis virus*	0.38	54.76	58.95	16.28	1.88	23.60	21.79	19.32	100.0

Table S5. Normalized percent relative abundance of detected eukaryotic viruses at the family level. Sum of rows = 100%

Viral Family	Week 0	Week 1	Week 2	Week 3	Week 4	Week 5	Week 6	Week 7
Poxviridae	0.00	0.00	0.00	0.00	0.00	0.00	0.00	0.00
Herpesviridae	24.86	0.00	5.30	37.27	21.54	10.16	0.87	0.00
Adenoviridae	0.00	0.00	0.00	0.00	0.00	0.00	88.84	11.16
Hepeviridae	0.00	0.00	0.00	0.00	0.00	0.00	0.00	0.00
Hepadnaviridae	0.00	0.00	0.00	0.00	0.00	0.00	0.00	0.00
Genomiviridae	0.00	0.00	0.00	0.00	0.00	0.00	0.00	0.00
Circoviridae	0.00	0.00	0.00	0.00	57.97	7.88	10.11	24.04
Parvoviridae	0.00	0.00	0.00	0.00	0.00	0.00	0.00	0.00
Reoviridae	0.00	0.00	0.00	0.00	0.00	0.00	0.00	0.00
Orthomyxoviridae	0.00	0.00	0.00	0.00	0.00	0.00	0.00	0.00
Phenumoviridae	0.00	0.00	0.00	0.00	0.00	0.00	0.00	0.00
Birnaviridae	0.00	0.00	0.00	0.00	0.32	0.00	95.39	4.28
Pneumoviridae	0.00	0.00	0.00	0.00	0.00	0.00	0.00	0.00
Paramyxoviridae	0.00	0.00	0.00	0.00	0.00	0.00	0.00	0.00
Astroviridae	0.00	100.00	0.00	0.00	0.00	0.00	0.00	0.00
Picornoviridae	0.00	0.00	0.00	97.06	2.94	0.00	0.00	0.00
Retroviridae	25.76	11.53	10.63	21.58	2.41	16.65	1.84	9.61
Flaviviridae	0.00	0.00	0.00	0.00	0.00	0.00	0.00	0.00
Coronaviridae	0.19	27.80	29.93	8.26	0.96	11.98	11.06	9.81

Table S6. Average relative abundance of detected bacteria.

Phylum		Class		Order		Family		Genus	
Actinobacteria	24.00%	Actinobacteria	24.00%	Corynebacteriales	5.25%	Corynebacteriaceae	5.25%		5.25%
				Micrococcales	13.53%	Brevibacteriaceae	5.67%	Brevibacterium	5.67%
						Dermabacteraceae	5.00%	Brachybacterium	5.00%
						Micrococcaceae	2.87%	Yaniella	0.87%
									2.00%
				Streptosporangiales	5.22%	Nocardiopsaceae	5.22%	Nocardiopsis	5.22%
Bacteroidetes	5.78%	Bacteroidia	3.38%	Bacteroidales	3.38%	Bacteroidaceae	2.33%	Bacteroides	2.33%
						Prevotellaceae	1.05%	Alloprevotella	1.05%
		Flavobacteria	2.40%	Flavobacteriales	2.40%	Flavobacteriaceae	2.40%	Chryseobacterium	2.40%
Firmicutes	56.17%	Bacilli	49.02%	Bacillales	21.35%	Bacillaceae	7.87%	Lentibacillus	0.83%
								Paucisalibacillus	1.93%
									5.10%
						Staphylococcaceae	11.43%	Jeotgalicoccus	0.68%
								Salinicoccus	2.07%
								Staphylococcus	8.68%
						Planococcaceae	2.05%		2.05%
				Lactobacillales	27.67%	Aerococcaceae	0.60%	Facklamia	0.60%
						Lactobacillaceae	14.50%	Lactobacillus	14.50%
						Leuconostocaceae	1.10%	Weissella	1.10%
						Streptococcaceae	2.03%	Streptococcus	2.03%
						Lactobacillales	8.28%		8.28%
									1.16%
		Clostridia	7.15%	Clostridiales	7.15%	Lachnospiraceae	2.59%	Lachnoclostridium	1.83%

									0.77%
						Ruminococcaceae	3.98%	Anaerotruncus	0.60%
								Faecalibacterium	3.00%
								Subdoligranulum	0.38%
						Peptostreptococcaceae	0.58%		0.58%
Proteobacteria	39.28%	Betaproteobacteria	3.00%	Burkholderiales	3.00%	Oxalobacteraceae	3.00%		3.00%
		Gammaproteobacteria	36.28%	Pasteurellales	27.33%	Pasteurellaceae	27.33%	Gallibacterium	27.33%
				Pseudomonadales	5.03%	Pseudomonadaceae	5.03%	Pseudomonas	5.03%
				Xanthomonadales	3.00%	Xanthomonadaceae	3.00%	Xanthomonas	3.00%
				Enterobacterales	0.92%	Enterobacteriaceae	0.92%	Escherichia-Shigella	0.92%

Table S7. Relative abundance of detected bacteria at the genera level. (Taxa that could not be assigned a genus are displayed using the highest taxonomic level that could be assigned to them: * (family), ** (class), or *** (order))

Family	Genus	Week 0	Week 1	Week 3	Week 4	Week 5	Week 7	Frequency
Corynebacteriaceae	Corynebacteriaceae*	6.00	1.00	1.00	1.00	0.50	22.00	100
Brevibacteriaceae	*Brevibacterium*	9.00	6.00	7.00	5.00	4.00	3.00	100
Dermabacteraceae	*Brachybacterium*	8.00	6.00	5.00	7.00	2.00	2.00	100
Micrococcaceae	*Yaniella*	1.00	1.00	1.00	1.00	1.00	0.20	100
	Micrococcaceae*	2.00	0.00	0.00	0.00	0.00	0.00	16.7
Nocardiopsaceae	*Nocardiopsis*	3.00	1.00	1.00	21.00	0.10	0.00	83.3
Bacteroidaceae	*Bacteroides*	0.00	0.00	3.00	2.00	2.00	0.00	50
Prevotellaceae	*Alloprevotella*	2.00	0.00	0.00	0.00	0.10	0.00	33.3
Flavobacteriaceae	*Chryseobacterium*	7.00	0.00	0.00	0.10	0.10	0.00	50
Bacillaceae	*Lentibacillus*	2.00	0.40	0.10	0.00	0.00	0.00	50
	Paucisalibacillus	1.00	2.00	2.00	6.00	0.30	0.30	100
	Jeotgalicoccus	1.00	0.40	0.30	1.00	0.40	1.00	100
Staphylococcaceae	*Salinicoccus*	1.00	2.00	2.00	4.00	1.00	2.40	100
	Staphylococcus	3.00	7.10	11.00	16.00	6.00	9.00	100
	Bacillaceae*	6.50	6.30	4.20	10.40	1.20	2.00	100
Planococcaceae	Planococcaceae*	4.00	0.00	0.00	0.00	0.10	0.00	33.3
Aerococcaceae	*Facklamia*	2.00	0.00	0.10	0.20	0.00	0.10	66.7
Lactobacillaceae	*Lactobacillus*	5.10	14.10	34.10	7.00	18.20	8.50	100
Leuconostocaceae	*Weissella*	0.00	1.00	0.40	3.00	1.00	0.10	83.3
Streptococcaceae	*Streptococcus*	8.00	1.10	0.10	0.40	0.40	2.20	100
Lactobacillales	Lactobacillales**	1.00	23.00	0.10	0.00	11.00	6.30	83.3
	Lachnoclostridium	0.00	3.00	2.00	2.00	0.30	0.00	66.7
Lachnospiraceae	*Anaerotruncus*	0.00	1.00	1.00	0.10	0.30	0.00	66.7
	Faecalibacterium	0.00	0.00	5.00	2.00	4.00	1.00	66.7
Ruminococcaceae	*Subdoligranulum*	0.00	0.00	1.00	0.20	0.20	0.10	66.7

	Lachnospiraceae*	0.00	2.00	0.20	0.10	0.00	0.00	50
	Peptostreptococcaceae*	0.00	1.00	0.10	1.00	0.40	0.40	83.3
Peptostreptococcaceae	Bacilli***	0.50	1.00	2.40	1.40	0.50	0.00	83.3
Oxalobacteraceae	Oxalobacteraceae*	3.00	0.00	0.00	0.00	0.00	0.00	16.7
Pasteurellaceae	*Gallibacterium*	0.00	0.00	1.00	0.00	44.00	37.00	50
Pseudomonadaceae	*Pseudomonas*	13.00	2.00	0.00	0.00	0.10	0.00	50
Xanthomonadaceae	*Xanthomonas*	3.00	0.00	0.00	0.00	0.00	0.00	16.7
Enterobacteriaceae	*Escherichia-Shigella*	0.10	2.00	1.00	2.00	0.20	0.20	100

Table S8. Average normalized relative abundance of detected bacteriophage.

Order		Family		Genus		Species	Average
Caudovirales	142.34%	Myoviridae	70.99%	P1virus	1.83%	*Enterobacteria phage P1*	0.75%
						Salmonella phage SJ46	1.08%
				Spounavirinae	6.97%	*Staphylococcus phage GH15*	0.67%
						Staphylococcus phage MCE-2014	1.29%
						Staphylococcus phage P108	1.13%
						Staphylococcus phage phiIPLA-RODI	2.50%
						Staphylococcus phage phiSA012	1.39%
				Tevenvirinae	39.89%	*Enterobacteria phage RB55*	39.16%
						Shigella phage SHFML-11	0.73%
				unclassified Myoviridae	22.31%	*Enterobacteria phage P88*	2.47%
						Enterobacteria phage phi92	2.78%
						Enterobacteria phage SfI	2.09%
						Salmonella phage RE-2010	6.53%
						Shigella phage SfIV	8.44%
		Podoviridae	40.19%	Autographivirinae	6.75%	*Enterobacteria phage T7*	6.75%
				Epsilon15virus	8.23%	*Escherichia phage TL-2011b*	8.23%
				Sepvirinae	24.15%	*Enterobacteria phage VT2phi_272*	4.10%
						Stx2 converting phage vB_EcoP_24B	20.05%
				Unclassified Podoviridae	1.06%	*Enterobacteria phage IME10*	1.06%
		Siphoviridae	31.16%	Lambdavirus	11.15%	*Enterobacteria phage cdtI*	1.98%
						Enterobacteria phage lambda	2.55%
						Enterobacteria phage mEp460	2.63%
						Stx2-converting phage 1717	3.99%
				Spbetavirus	0.50%	*Staphylococcus phage SPbeta-like*	0.50%
				unclassified Siphoviridae	19.51%	*Lactobacillus prophage Lj928*	6.35%
						Microbacterium phage Min1	2.51%
						Staphylococcus phage phiRS7	1.84%
						Staphylococcus phage StB20	4.79%
						Staphylococcus phage StB20-like	4.02%
Unclassified	4.83%	Unclassified	4.83%	Unclassified	4.83%	*Enterobacteria phage YYZ-2008*	4.25%
						Uncultured phage crAssphage	0.59%

Table S9. Normalized abundance of detected bacteriophage at the species level.

Species	Week 0	Week 1	Week 2	Week 3	Week 4	Week 5	Week 6	Week 7
Enterobacteria phage IME10	202.28	1258.09	150.16	611.33	0.00	0.00	0.00	0.00
Staphylococcus phage SPbeta-like	313.94	0.00	0.00	284.63	90.51	0.00	0.00	0.00
Enterobacteria phage P88	0.00	928.47	0.00	1353.48	484.17	0.00	0.00	0.00
Staphylococcus phage GH15	0.00	59.46	0.00	1040.16	41.34	0.00	0.00	0.00
Shigella phage SHFML-11	140.99	97.43	69.77	0.00	33.87	160.41	0.00	70.65
Enterobacteria phage P1	253.79	613.83	62.80	894.81	0.00	0.00	0.00	0.00
Staphylococcus phage StB20-like	0.00	0.00	0.00	3873.58	568.48	168.27	0.00	0.00
Staphylococcus phage P108	0.00	0.00	0.00	1290.95	123.15	48.60	0.00	0.00
Staphylococcus phage phiSA012	0.00	0.00	0.00	1108.69	244.06	48.16	0.00	0.00
Microbacterium phage Min1	2075.63	896.48	0.00	0.00	0.00	0.00	412.69	0.00
Staphylococcus phage phiRS7	924.06	191.57	0.00	3071.89	133.20	315.42	0.00	0.00
Enterobacteria phage cdtI	170.56	2298.33	0.00	773.17	245.85	0.00	135.64	128.21
Enterobacteria phage SfI	1462.35	1732.38	310.15	1578.36	0.00	178.27	0.00	0.00
Staphylococcus phage StB20	0.00	0.00	0.00	2979.68	1421.19	168.27	156.82	148.23
Salmonella phage SJ46	387.63	1285.79	57.55	937.18	223.50	132.32	61.66	0.00
Staphylococcus phage MCE-2014	56.51	58.58	0.00	2732.68	0.00	144.68	0.00	42.48
Enterobacteria phage lambda	2480.22	2399.55	122.74	249.85	476.68	141.10	526.00	0.00
Enterobacteria phage phi92	0.00	55.94	0.00	978.52	933.43	184.20	0.00	0.00
Enterobacteria phage mEp460	2702.67	3175.06	267.49	1089.04	0.00	461.27	143.29	0.00
Lactobacillus prophage Lj928	0.00	10612.23	465.28	0.00	150.58	0.00	0.00	0.00
Staphylococcus phage phiIPLA-RODI	56.34	0.00	0.00	3490.40	284.23	480.77	0.00	42.35
Salmonella phage RE-2010	9637.67	4873.26	523.47	4617.59	847.08	802.38	186.95	353.40
Enterobacteria phage T7	7630.75	5620.16	298.12	3944.67	434.18	856.81	798.51	452.84
Stx2-converting phage 1717	5032.73	4012.93	478.95	2729.93	186.01	550.60	205.26	194.00
Enterobacteria phage VT2phi_272	6809.25	3781.24	270.78	3123.52	438.18	518.81	386.81	91.40
Shigella phage SfIV	11295.95	7318.20	898.40	3962.43	436.14	860.67	1283.37	0.00
Escherichia phage TL-2011b	11998.06	7981.90	398.79	4600.12	1032.51	916.89	996.93	269.22
Enterobacteria phage RB55	0.00	0.00	3982.75	0.00	0.00	5388.95	0.00	1284.90
Stx2 converting phage vB_EcoP_24B	38098.43	20466.63	825.71	12816.52	2004.26	949.24	3096.30	522.60
Uncultured phage crAssphage	1487.20	171.29	0.00	124.85	0.00	0.00	0.00	0.00
Enterobacteria phage YYZ-2008	5989.66	3482.95	108.44	2428.26	526.45	0.00	697.11	109.82

Table S10. Normalized percent relative abundance of detected bacteriophage at the species level.

Family	Species	Week 0	Week 1	Week 2	Week 3	Week 4	Week 5	Week 6	Week 7	Frequency
Myoviridae	*Enterobacteria phage P88*		1.114		2.030	4.262				50
	Staphylococcus phage GH15		0.071		1.560	0.364				37.5
	Shigella phage SHFML-11	0.129	0.117	0.751	0.000	0.298	1.190		1.904	75
	Enterobacteria phage P1	0.232	0.736	0.676	1.342					50
	Staphylococcus phage P108				1.936	1.084	0.361			37.5
	Staphylococcus phage phiSA012				1.663	2.149	0.357			37.5
	Enterobacteria phage SfI	1.339	2.078	3.338	2.367	0.000	1.323			62.5
	Salmonella phage SJ46	0.355	1.542	0.619	1.405	1.968	0.982	0.678		87.5
	Staphylococcus phage MCE-2014	0.052	0.070		4.098		1.074		1.145	62.5
	Enterobacteria phage phi92		0.067		1.467	8.218	1.367			50
	Staphylococcus phage phiIPLA-RODI	0.052			5.234	2.502	3.568		1.141	62.5
	Salmonella phage RE-2010	8.825	5.845	5.634	6.924	7.457	5.954	2.057	9.525	100
	Shigella phage SfIV	10.344	8.778	9.669	5.942	3.840	6.387	14.123		87.5
	Enterobacteria phage RB55			42.865			39.989		34.632	37.5
Podoviridae	*Enterobacteria phage IME10*	0.185	1.509	1.616	0.917					50
	Enterobacteria phage T7	6.987	6.741	3.209	5.915	3.822	6.358	8.787	12.206	100
	Enterobacteria phage VT2phi_272	6.235	4.535	2.914	4.684	3.858	3.850	4.257	2.464	100
	Escherichia phage TL-2011b	10.987	9.574	4.292	6.898	9.090	6.804	10.970	7.256	100
	Stx2 converting phage vB_EcoP_24B	34.887	24.549	8.887	19.219	17.645	7.044	34.073	14.086	100
Siphoviridae	*Staphylococcus phage SPbeta-like*	0.287			0.427	0.797				37.5
	Staphylococcus phage StB20-like				5.809	5.005	1.249			37.5
	Microbacterium phage Min1	1.901	1.075					4.541		37.5
	Staphylococcus phage phiRS7	0.846	0.230		4.606	1.173	2.341			62.5
	Enterobacteria phage cdtI	0.156	2.757		1.159	2.164		1.493	3.456	75
	Staphylococcus phage StB20				4.468	12.512	1.249	1.726	3.995	62.5
	Enterobacteria phage lambda	2.271	2.878	1.321	0.375	4.196	1.047	5.788		87.5
	Enterobacteria phage mEp460	2.475	3.808	2.879	1.633		3.423	1.577		75
	Lactobacillus prophage Lj928	0.000	12.729	5.008		1.326				37.5

	Stx2-converting phage 1717	4.608	4.813	5.155	4.094	1.638	4.086	2.259	5.229	100
Unclassified	*Uncultured phage crAssphage*	1.362	0.205		0.187					37.5
	Enterobacteria phage YYZ-2008	5.485	4.178	1.167	3.641	4.635		7.671	2.960	87.5

Table S11. Average normalized relative abundance of detected fungi.

Phylum		Class		Order		Family		Genus		Species	Average Abundance
Ascomycota	103.8 %	Dothideomycetes	0.15%	Capnodiales	0.05%	Mycosphaerellaceae	0.05%	Zymoseptoria	0.05%	*Bipolaris cookei*	0.02%
										Didymella pinodes	0.03%
				Pleosporales	0.09%	Astrosphaeriellaceae	0.04%	Pithomyces	0.04%	*Mycosphaerella graminicola*	0.04%
						Didymellaceae	0.03%	Didymella	0.03%	*Pithomyces chartarum*	0.03%
						Pleosporaceae	0.03%	Bipolaris	0.02%	*Stemphylium lycopersici*	0.02%
								Stemphylium	0.00%	*Zymoseptoria tritici*	0.002%
		Eurotiomycetes	0.99%	Onygenales	0.08%	Arthrodermataceae	0.08%	Trichophyton	0.08%	*Aspergillus fumigatus*	0.08%
				Chaetothyriales	0.06%	Herpotrichiellaceae	0.06%	Exophiala	0.06%	*Aspergillus nidulans*	0.06%
				Eurotiales	0.86%	Aspergillaceae	0.45%	Aspergillus	0.32%	*Aspergillus oryzae*	0.01%
						Aspergillaceae				*Epidermophyton floccosum*	0.28%
										Exophiala dermatitidis	0.04%
								Penicillium	0.13%	*Penicillium chrysogenum*	0.13%
						Trichocomaceae	0.41%	Talaromyces	0.41%	*Talaromyces marneffei*	0.41%
				Onygenales	0.54%	Arthrodermataceae	0.54%	Epidermophyton	0.54%	*Trichophyton rubrum*	0.54%
		Lecanoromycetes	0.55%	Lecanorales	0.01%	Parmeliaceae	0.01%	Usnea	0.01%	*Ricasolia amplissima*	0.01%
				Peltigerales	0.03%	Lobariaceae	0.03%	Ricasolia	0.03%	*Usnea ceratina*	0.03%
		Leotiomycetes	0.01%	Helotiales	0.01%	Sclerotiniaceae	0.01%	Botrytis	0.01%	*Amorphotheca resinae*	0.01%
				Leotiomycetes incertae sedis	0.00%	Myxotrichaceae	0.00%	Amorphotheca	0.00%	*Botrytis cinerea*	0.003%
		Saccharomycetes	98.76 %	Saccharomycetales	98.76 %	Debaryomycetaceae	0.02%	Candida	0.01%	*Candida dubliniensis*	0.01%
								Debaryomyces	0.01%	*Candida glabrata*	0.01%
								Meyerozyma	0.00%	*Debaryomyces hansenii*	0.01%
						Dipodascaceae	0.03%	Yarrowia	0.03%	*Dekkera bruxellensis*	0.03%

				Phaffomycetaceae	0.01%	Wickerhamomyces	0.01%	*Eremothecium gossypii*	0.01%
				Pichiaceae	0.02%	Brettanomyces	0.02%	*Eremothecium sinecaudum*	0.02%
				Saccharomycetaceae	0.54%	Eremothecium	0.01%	*Kazachstania Naganishii*	0.01%
								Kluyveromyces marxianus	0.001%
						Kazachstania	0.01%	*Kluyveromyces lactis*	0.002%
								Kuraishia capsulata	0.01%
						Kluyveromyces	0.01%	*Lachancea thermotolerans*	0.01%
						Lachancea	0.46%	*Meyerozyma guilliermondii*	0.47%
						Nakaseomyces	0.00%	*Saccharomyces cerevisiae*	0.002%
						Saccharomyces	0.00%	*Sugiyamaella lignohabitans*	0.004%
						Tetrapisispora	0.05%	*Tetrapisispora blattae*	0.001%
								Tetrapisispora phaffii	0.04%
						Torulaspora	0.01%	*Torulaspora delbrueckii*	0.01%
				Sacc. Incertae sedis	98.14%	Kuraishia	98.14%	*Wickerhamomyces ciferrii*	98.14%
				Trichomonascaceae	0.00%	Sugiyamaella	0.00%	*Yarrowia lipolytica*	0.002%
Schizosaccharomycetes	0.00%	Schizosaccharomycetales	0.00%	Schizosaccharomycetaceae	0.00%	Schizosaccharomyces	0.00%	*Schizosaccharomyces pombe*	0.002%
Sordariomycetes	3.37%	Diaporthales	0.10%	Cryphonectriaceae	0.06%	Chrysoporthe	0.06%	*Chrysoporthe austroafrica*	0.04%
								Chrysoporthe deuterocubensis	0.02%
				Diaporthaceae	0.04%	Diaporthe	0.04%	*Clonostachys rosea*	0.05%
		Glomerellales	0.52%	Glomerellaceae	0.52%	Colletotrichum	0.52%	*Colletotrichum graminicola*	0.52%
		Hypocreales	2.19%	Bionectriaceae	1.47%	Clonostachys	1.47%	*Diaporthe longicolla*	1.47%
				Hypocreaceae	0.22%	Trichoderma	0.22%	*Fusarium circinatum*	0.22%
				Nectriaceae	0.50%	Fusarium	0.41%	*Fusarium graminearum*	0.13%
								Fusarium mangiferae	0.22%
								Gibberella moniliformis	0.04%

										Nectria cinnabarina	0.03%
								Nectria	0.08%	*Neurospora crassa*	0.09%
				Microascales	0.05%	Microascaceae	0.05%	Scedosporium	0.05%	*Pestalotiopsis fici*	0.05%
				Sordariales	0.03%	Chaetomiaceae	0.02%	Thermothelomyces	0.01%	*Scedosporium apiospermum*	0.01%
								Thielavia	0.01%	*Sordaria macrospora*	0.02%
						Sordariaceae	0.00%	Neurospora	0.00%	*Thermothelomyces thermophila*	0.002%
								Sordaria	0.00%	*Thielavia terrestris*	0.002%
				Xylariales	0.48%	Sporocadaceae	0.48%	Pestalotiopsis	0.48%	*Trichoderma asperellum*	0.48%
Basidiomycota	0.08%	Tremellomycetes	0.03%	Agaricales	0.03%	Agaricaceae	0.00%	Agaricus	0.00%	*Cryptococcus neoformans*	0.002%
						Marasmiaceae	0.00%	Moniliophthora	0.00%	*Cryptococcus gattii*	0.003%
						Tricholomataceae	0.03%	Laccaria	0.03%	*Tremella fuciformis*	0.03%
		Agaricomycetes	0.05%	Tremellales	0.05%	Cryptococcaceae	0.02%	Cryptococcus	0.02%	*Agaricus bisporus*	0.01%
										Laccaria bicolor	0.02%
						Tremellaceae	0.03%	Tremella	0.03%	*Moniliophthora perniciosa*	0.03%

Table S12. Normalized abundance of detected fungi at the species level.

Species	Week 0	Week 1	Week 2	Week 3	Week 4	Week 5	Week 6	Week 7
Candida glabrata	4.53				3.79	11.63		
Agaricus bisporus					8.73	2.99		
Amorphotheca resinae						3.17	5.13	
Aspergillus fumigatus		183.48		170.82				
Aspergillus nidulans		169.51						
Aspergillus oryzae	1.18	25.15	1.73	9.14	2.96	1.83	1.03	4.35
Bipolaris cookei						43.02		
Botrytis cinerea	4.84	2.87	2.94	2.43	2.93	2.70		
Candida dubliniensis		2.48			9.31		1.78	2.36
Chrysoporthe austroafricana		177.08		27.48				
Chrysoporthe deuterocubensis				42.15				
Clonostachys rosea		137.64						
Colletotrichum graminicola				925.76				
Cryptococcus gattii						2.94	2.45	
Cryptococcus neoformans		3.74		3.75				
Debaryomyces hansenii	3.69	7.92		8.80	4.07			
Dekkera bruxellensis		73.67		68.59				
Diaporthe longicolla		18760.22	125.00	1177.48	93.52	109.31		
Didymella pinodes	178.10							
Epidermophyton floccosum			216.10	169.64				
Eremothecium gossypii					5.51	3.24		
Eremothecium sinecaudum					5.48			
Exophiala dermatitidis	191.68							
Fusarium circinatum		587.48		703.22		174.09		

Fusarium graminearum		58.87		438.45				
Fusarium mangiferae				171.20				352.62
Gibberella moniliformis	92.73			97.55				
Kazachstania naganishii				5.28	3.87			
Kluyveromyces lactis						3.41		
Kluyveromyces marxianus		3.71		3.06				
Kuraishia capsulata	4.41	4.99			4.42			
Laccaria bicolor	4.83	9.98	3.31	4.87	2.48	7.43	4.03	9.27
Lachancea thermotolerans					3.28	3.40		
Meyerozyma guilliermondii					160.86			
Moniliophthora perniciosa				48.06				
Mycosphaerella graminicola	226.75							
Nectria cinnabarina		80.58						
Neurospora crassa		260.59						
Penicillium chrysogenum	89.75	42.56	6.87	45.95	288.28	147.93	15.96	6.87
Pestalotiopsis fici	286.75							
Pithomyces chartarum	144.63							
Ricasolia amplissima	60.54							
Saccharomyces cerevisiae		6.81						
Scedosporium apiospermum						14.31		
Schizosaccharomyces pombe								2.20
Sordaria macrospora	4.85	6.41	10.35		2.89		6.32	22.04
Stemphylium lycopersici		74.19						
Sugiyamaella lignohabitans		4.37		7.41	1.80			
Talaromyces marneffei				147.97				761.92
Tetrapisispora blattae	6.77			1.94				
Tetrapisispora phaffii		68.48	45.12	21.25	20.25	23.67	26.32	58.36
Thermothelomyces thermophila	1.30	1.46	1.48	1.06	0.46	1.63	1.29	1.94
Thielavia terrestris	1.98	1.37	1.92	1.49	1.23	2.20	1.00	0.90

Torulaspora delbrueckii							4.00	
Tremella fuciformis	142.18							
Trichoderma asperellum					166.60			
Trichophyton rubrum			247.54					
Usnea ceratina		85.94				89.13		
Wickerhamomyces ciferrii	539341.10	286941.40	45174.40	174945.06	33799.87	218599.53	50508.57	51128.68
Yarrowia lipolytica	2.16	1.37		1.44	2.17	1.61	1.49	
Zymoseptoria tritici	1.42				1.43			

Table S13. Normalized percent relative abundance of detected fungi at the species level.

Species	Week 0	Week 1	Week 2	Week 3	Week 4	Week 5	Week 6	Week 7	Frequency
Candida glabrata	0.0008				0.0109	0.0053			37.5
Agaricus bisporus					0.0252	0.0014			25
Amorphotheca resinae						0.0014	0.0101		25
Aspergillus fumigatus		0.0596		0.0953					25
Aspergillus nidulans		0.0551							12.5
Aspergillus oryzae	0.0002	0.0082	0.0038	0.0051	0.0086	0.0008	0.0020	0.0126	87.5
Bipolaris cookei						0.0196			12.5
Botrytis cinerea	0.0009	0.0009	0.0064	0.0014	0.0085	0.0012			75
Candida dubliniensis		0.0008			0.0269		0.0035	0.0023	50
Chrysoporthe austroafricana		0.0575		0.0153					25
Chrysoporthe deuterocubensis				0.0200					12.5
Clonostachys rosea		0.0447							12.5
Colletotrichum graminicola				0.5165					12.5
Cryptococcus gattii						0.0013	0.0049		25
Cryptococcus neoformans		0.0012		0.0021					25
Debaryomyces hansenii	0.0007	0.0026		0.0049	0.0118				50
Dekkera bruxellensis		0.0239		0.0383					25
Diaporthe longicolla		6.0952	0.2727	0.6569	0.2703	0.0499			62.5
Didymella pinodes	0.0329								12.5
Epidermophyton floccosum			0.4715	0.0946					25
Eremothecium gossypii					0.0159	0.0015			25
Eremothecium sinecaudum					0.0158				12.5
Exophiala dermatitidis	0.0354								12.5
Fusarium circinatum		0.1909		0.3923		0.0794			37.5
Fusarium graminearum		0.0191		0.2446					25

Fusarium mangiferae				0.0955				0.3405	25
Gibberella moniliformis	0.0171			0.0544					25
Kazachstania naganishii				0.0029	0.0112				25
Kluyveromyces lactis						0.0016			12.5
Kluyveromyces marxianus		0.0012		0.0017					25
Kuraishia capsulata	0.0008	0.0016			0.0128				37.5
Laccaria bicolor	0.0009	0.0032	0.0072	0.0027	0.0072	0.0034	0.0080	0.0179	100
Lachancea thermotolerans					0.0095	0.0015			25
Meyerozyma guilliermondii					0.4650				12.5
Moniliophthora perniciosa				0.0268					12.5
Mycosphaerella graminicola	0.0419								12.5
Nectria cinnabarina		0.0262							12.5
Neurospora crassa		0.0847							12.5
Penicillium chrysogenum	0.0166	0.0138	0.0150	0.0256	0.8333	0.0675	0.0315	0.0265	100
Pestalotiopsis fici	0.0530								12.5
Pithomyces chartarum	0.0267								12.5
Ricasolia amplissima	0.0112								12.5
Saccharomyces cerevisiae		0.0022							12.5
Scedosporium apiospermum						0.0065			12.5
Schizosaccharomyces pombe								0.0021	12.5
Sordaria macrospora	0.0009	0.0021	0.0226		0.0084		0.0125	0.0426	75
Stemphylium lycopersici		0.0241							12.5
Sugiyamaella lignohabitans		0.0014		0.0041	0.0052				37.5
Talaromyces marneffei				0.0825				0.7358	25
Tetrapisispora blattae	0.0013			0.0011					25
Tetrapisispora phaffii		0.0222	0.0984	0.0119	0.0585	0.0108	0.0520	0.0564	87.5
Thermothelomyces thermophila	0.0002	0.0005	0.0032	0.0006	0.0013	0.0007	0.0026	0.0056	75
Thielavia terrestris	0.0004	0.0004	0.0042	0.0008	0.0035	0.0010	0.0020	0.0026	75
Torulaspora delbrueckii							0.0079		12.5

Tremella fuciformis	0.0263								12.5
Trichoderma asperellum					0.4816				12.5
Trichophyton rubrum			0.5400						12.5
Usnea ceratina		0.0279				0.0407			12.5
Wickerhamomyces ciferrii	99.7309	93.2272	98.5550	97.5976	97.6982	99.7037	99.8600	98.7550	100
Yarrowia lipolytica	0.0004	0.0004		0.0008	0.0063	0.0007	0.0029		50
Zymoseptoria tritici	0.0003				0.0041				12.5
